# *Staphylococcus aureus* Nasal Colonization: An Update on Mechanisms, Epidemiology, Risk Factors, and Subsequent Infections

**DOI:** 10.3389/fmicb.2018.02419

**Published:** 2018-10-08

**Authors:** Adèle Sakr, Fabienne Brégeon, Jean-Louis Mège, Jean-Marc Rolain, Olivier Blin

**Affiliations:** ^1^Faculté de Médecine et de Pharmacie, IRD, APHM, MEPHI, IHU Méditerranée Infection, Aix-Marseille Université, Marseille, France; ^2^Service de Pharmacologie Clinique et Pharmacovigilance, AP-HM, Pharmacologie Intégrée et Interface Clinique et Industriel, Institut des Neurosciences Timone – UMR AMU-INSERM 1106, Aix-Marseille Université, Marseille, France

**Keywords:** *Staphylococcus aureus*, nasal colonization, epidemiology, surgical site infections (SSI), nasal microbiota, predisposing factors, nasal carriage

## Abstract

Up to 30% of the human population are asymptomatically and permanently colonized with nasal *Staphylococcus aureus*. To successfully colonize human nares, *S. aureus* needs to establish solid interactions with human nasal epithelial cells and overcome host defense mechanisms. However, some factors like bacterial interactions in the human nose can influence *S. aureus* colonization and sometimes prevent colonization. On the other hand, certain host characteristics and environmental factors can predispose to colonization. Nasal colonization can cause opportunistic and sometimes life-threatening infections such as surgical site infections or other infections in non-surgical patients that increase morbidity, mortality as well as healthcare costs.

## Introduction

*Staphylococcus aureus* is both a human skin and mucosae commensal but also a frequent cause of serious infections with high morbidity, mortality, and healthcare-associated costs (Schmidt et al., 2015). The most frequent carriage site is the *vestibulum nasi* (or anterior nares), which serves as reservoir for the spread of the pathogen (Williams, 1963; Sivaraman et al., 2009). This bacteria can establish solid interactions with nasal epithelial cells via various proteins and many cell surface components (Wertheim et al., 2005a; Mulcahy and McLoughlin, 2016), thus transforming into persistent carriage. *S. aureus* colonizes the anterior nares of 20% to 80% of the human population (Brown et al., 2014). Nasal carriage has been shown to play a key role in the pathogenesis of *S. aureus* infections (Kluytmans et al., 1997) in patients undergoing surgery (Perl et al., 2002; Bode et al., 2010), dialysis (Kluytmans et al., 1996; Nouwen et al., 2006), and in intensive care unit (ICU) patients (Garrouste-Orgeas et al., 2001), with higher infection risks in persistent carriers (Nouwen et al., 2006).

Previously published reviews on *S. aureus* carriage have usually focused independently on colonization or infections, or have issued a specific underlying condition or surgery. Here, we will full review recent advances in nasal microbiota composition and interspecies interactions, epidemiology, and risk factors for *S. aureus* colonization as well as the link between nasal carriage and infections both in community and nosocomial context.

## Nasal Microbiota and Interactions Between Bacteria

The adult nasal microbiota differs between individuals, but species belonging to *Corynebacterium, Propionibacterium*, and *Staphylococcus* genera are the most abundant bacteria (Frank et al., 2010; Human Microbiome Project Consortium, 2012; Yan et al., 2013; Kaspar et al., 2016). In a study conducted on the nasal microbiota of 178 adults, 88.2% were *Corynebacterium* carriers, 83.7% *Propionibacterium acnes* carriers, and 90.4% *Staphylococcus epidermidis* carriers. Proportional abundance varied considerably between individuals (Liu C.M. et al., 2015).

**FIGURE 1:**
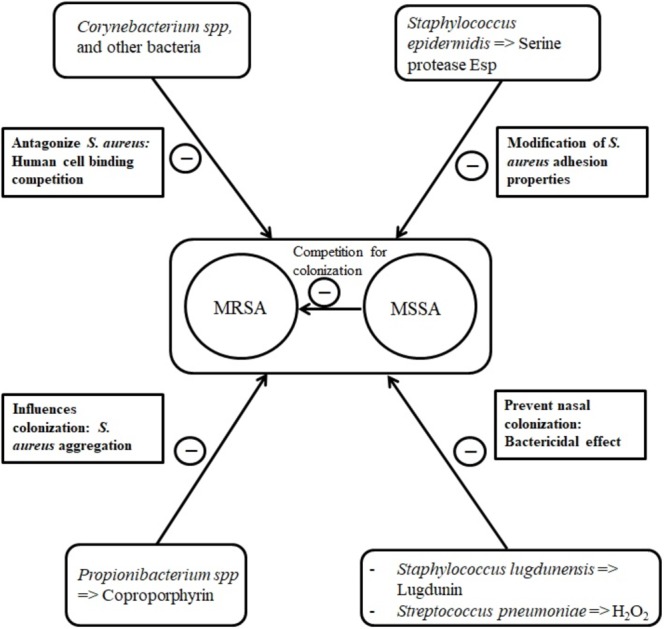
Main bacterial interactions with nasal *S. aureus*.

The health status may influence the nasal microbiota and vice versa. In a study involving healthy and hospitalized individuals, healthy adults harbored nares microbiota dominated by *Actinobacteria* (mainly *Propionibacterium* and *Corynebacterium* spp.) whereas patients microbiota were dominated by *S. aureus* and *S. epidermidis*. *S. aureus* colonization was negatively associated with the presence of other bacteria including *S. epidermidis* (Frank et al., 2010). Such counterweight effect between bacteria could be the result of interdependent activation-inhibition mechanisms as reviewed by Krismer et al. (2017). In fact, some bacterial species are capable of secreting anti-staphylococcal molecules modulating *S. aureus* abundance (**Figure 1**). For instance, *in vitro* production of H_2_O_2_ by *Streptococcus pneumoniae* can be bactericidal on *S. aureus* (Regev-Yochay et al., 2006; Selva et al., 2009). Recently, an *in vitro* and human study demonstrated that lugdunin, a non-ribosomal synthesized bioactive compound produced by *Staphylococcus lugdunensis*, can prevent *S. aureus* nasal colonization via a bactericidal effect (Zipperer et al., 2016).

In some cases, the bacteria-secreted molecules can modify *S. aureus* adhesion properties. Some types of *S. epidermidis* seem to be capable of synthetizing the serine protease Esp that eliminates nasal *S. aureus* in healthy humans (Iwase et al., 2010), probably by degrading staphylococcal surface proteins and human receptors critical for host–pathogen interaction (Sugimoto et al., 2013). As well, *Propionibacterium* species produce coproporphyrin III, a porphyrin metabolite that induces *S. aureus* aggregation which influences nasal colonization (Wollenberg et al., 2014).

*Corynebacterium* species are suggested to antagonize *S. aureus* by human cell binding competition mechanisms (Uehara et al., 2000; Lina et al., 2003). In 156 healthy volunteers, Uehara et al. (2000) observed a 71% total eradication rate of nasal *S. aureus* after performing up to 15 inoculations of a *Corynebacterium* sp. strain to the nares of *S. aureus* carriers.

Intra-species competition has also been described. In a cross-sectional clinical study, it was suggested that methicillin-sensitive *S. aureus* (MSSA) and methicillin-resistant *S. aureus* (MRSA) compete for colonization, MSSA being protective with regard to MRSA carriage (Dall’Antonia et al., 2005). On the other hand, pre-existing nasal carriage with *S. aureus* could predispose adult patients to further staphylococcal colonization (Ghasemzadeh-Moghaddam et al., 2015).

## Spread and Transmission of *S. aureus*

**FIGURE 2:**
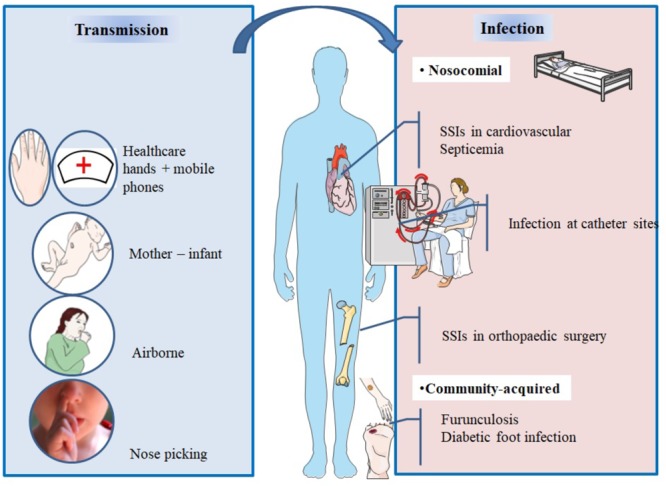
Main spread and transmission mechanisms of *S. aureus* and impact of nasal carriage on subsequent infections.

*Staphylococcus aureus* can be found in different body sites like the skin, rectum, vagina, gastrointestinal tract and axilla, the anterior nares appearing as the main reservoir. From a cutaneous commensal site, *S. aureus* can enter in contact with the nasal mucosa, then interact with epithelial cell ligands such as loricrin and cytokeratin 10 (K10) (**Table 1**). Once the host’s defenses are overcome, *S. aureus* can propagate into the anterior nares so that the host becomes an *S. aureus* nasal carrier (Wertheim et al., 2005a). In human, nasal colonization may begin within the first days of life (Maayan-Metzger et al., 2017). This has been demonstrated in a cohort study evaluating nasal carriage of *S. aureus* in 100 pairs of infant–mother for a period of 6 months following delivery (Peacock et al., 2003). The carriage rate in the first 8 weeks of life was around 40–50%, thereafter it dropped to 21% at 6 months. In addition, this study found a nasal carriage concordance in 68% of infant–mother pairs attesting the role of environmental factors in *S. aureus* carriage (Peacock et al., 2003). Another study found identical strains in 80% of infant–mother pairs. In 90% of these newborns, the source of *S. aureus* was the maternal nasal strain (Leshem et al., 2012; **Figure 2**).

**Table 1:** Major *S. aureus*-host ligands.

*S. aureus* ligand factor	Host ligand	Reference
ClfB	Loricrin, K10 (cytokeratin 10), K8 (cytokeratin 8), fibrinogen	Perkins et al., 2001; O’Brien et al., 2002; Schaffer et al., 2006; Wertheim et al., 2008; Haim et al., 2010; Mulcahy et al., 2012
IsdA	Fibrinogen, fibronectin	Clarke et al., 2004
SdrC	Unknown	Corrigan et al., 2009
SdrD	Desmoglein 1	Corrigan et al., 2009; Askarian et al., 2016
SasX	Unknown	Liu Q. et al., 2015
SasG	Unknown	Roche et al., 2003
WTA	Srec-1	Baur et al., 2014; Weidenmaier et al., 2004

After birth, hands are the main vector for *S. aureus* transmission from surfaces to the nose (Wertheim et al., 2005a). The hypothesis of a link between hand and nose *S. aureus* carriage is supported by the double blind randomized placebo controlled trial from Reagan et al. (1991), who demonstrated that nasal decolonization with mupirocin applied to health-care workers resulted in a decrease of nose and hand carriage. In a cohort study including outpatients and healthy hospital employees, nasal carriage was evaluated by a single or several swabs. Participants completed a questionnaire about their nose picking behavior, a positive correlation between this habit and nasal carriage of *S. aureus* was found. However, it is unknown whether nose-picking patients were more frequently colonized at extra nasal sites (Wertheim et al., 2006).

Studies realized in individuals living in the same households have revealed that these people tend to carry genetically similar strains in their nares (Nouwen and Optima Grafische Communicatie, 2004; Muthukrishnan et al., 2013) suggesting horizontal transmission. Multisite MRSA carriage increases the risk for nasal MRSA colonization (Harbarth et al., 2000).

In spite infrequent, airborne transmission is another possible route of *S. aureus* dissemination (Wertheim et al., 2005a) and may play a role in hospital outbreaks (Sherertz et al., 1996).

During viral upper respiratory infections, the risk of disseminating endogenous *S. aureus* in the air increases and infection outbreaks may occur. In 1996, an MRSA outbreak involving 8 of 43 patients occurred in a surgical ICU of a university hospital in the United States. Investigations of the cause concluded that a single physician was the source of this outbreak; he was a nasal carrier of MRSA and suffered an upper respiratory infection. To assess airborne dispersal of *S. aureus*, the authors completed their findings by an experimental clinical test on this physician and showed that transmission of the bacterium increased by 40-fold when he was infected by a rhinovirus infection than when he was not. The use of a mask significantly reduced dispersal (*P* = 0.015) (Sherertz et al., 1996).

Healthcare workers who are asymptomatic nasal carriers can sometimes be the source of MRSA outbreaks (Wang et al., 2001; Vonberg et al., 2006; Haill et al., 2013; Lamanna et al., 2017). On the other hand, in nonoutbreak situations and in presence of control measures, healthcare workers are infrequently sources of transmission of *S. aureus* (Price et al., 2017).

Mobile phones of healthcare workers may be a reservoir of *S. aureus* (Chang et al., 2017). A recent study evaluated incidence of bacterial contamination of mobile phones belonging to medical staff working in the operating room. Seventy two healthcare professionals took bacterial cultures from their phones, anterior nares, and hands. The results revealed that 31 staff had *S. aureus* isolated from their nares, 8 from their mobile phones, and 4 from their hands. Genotyping confirmed that 7/8 of the mobile phones strains were identical to the ones isolated from the nares (Chang et al., 2017).

## Mechanisms of Colonization

The *vestibulum nasi* or anterior part of the nares is lined by a stratified, keratinized nonciliated squamous epithelium, whereas the rest of the nasal cavity, that is, its inner part is lined with a ciliated columnar epithelium (Peacock et al., 2001; Weidenmaier et al., 2012).

**FIGURE 3:**
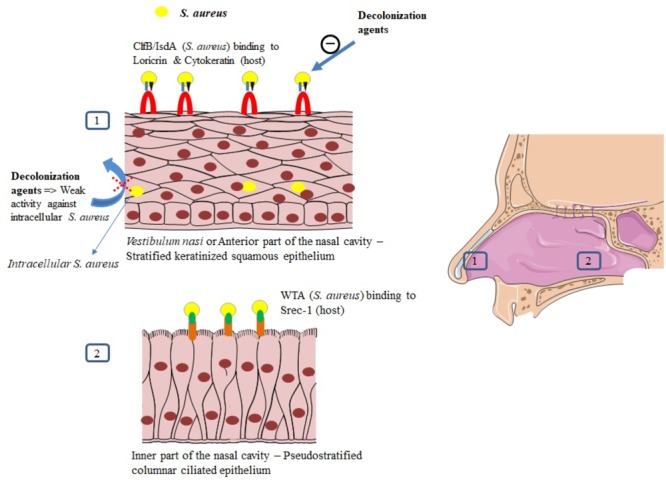
Mechanisms of *S. aureus* nasal colonization.

Both epithelia have been described as habitats for *S. aureus* (Mulcahy et al., 2012; Baur et al., 2014) as it will be developed in this section. Intracellular localization in nasal tissue from healthy volunteers was also described (Hanssen et al., 2017). For a successful colonization, *S. aureus* expresses adhesive molecules (Burian et al., 2010), fundamental for the establishment of interactions with human cell surface components, as it was demonstrated *in vitro* and *in vivo* (Mulcahy et al., 2012; Baur et al., 2014; **Figure 3**). Major ligands interactions are listed in **Table 1**.

## Interaction Between *S. aureus* and the Squamous Epithelium of the Anterior Nares

The *vestibulum nasi* is lined by skin (Cunningham, 1905; Vuyk and Watts, 2006). The uppermost layer of the epidermis is the *stratum corneum* or cornified layer (Candi et al., 2005; Eckhart et al., 2013). This layer contains keratinocytes that express proteins, such as loricrin, cytokeratin 10 (K10), involucrin, filaggrin, and other proteins (Eckhart et al., 2013).

Clumping factor B (ClfB) and iron-regulated surface determinant A (IsdA) are staphylococcal surface proteins that can adhere to cornified envelope proteins (Clarke et al., 2004, 2006; Schaffer et al., 2006; Wertheim et al., 2008; Mulcahy et al., 2012) and favor nasal colonization as it will be developed in this section.

Recently, Mulcahy et al. (2012) demonstrated that loricrin, the most abundant protein in the cornified envelope (Steinert and Marekov, 1995), was the major target ligand for ClfB during *S. aureus* nasal colonization. Nasal colonization by a ClfB^+^ *S. aureus* strain was reduced by 80% in loricrin-deficient mice compared to wild-type mice. ClfB has also been shown to interact with cytokeratin 10 (O’Brien et al., 2002; Schaffer et al., 2006; Wertheim et al., 2008), cytokeratin 8 (Haim et al., 2010), and fibrinogen (Perkins et al., 2001; **Table 1**). The role of ClfB in the adherence of *S. aureus* to the nasal epithelium has been studied *in vitro* (O’Brien et al., 2002; Mulcahy et al., 2012), in animal models (Schaffer et al., 2006; Mulcahy et al., 2012), and in human studies (Wertheim et al., 2008).

Schaffer et al. (2006) demonstrated that mutant strains of *S. aureus* deficient in ClfB resulted in a reduced nasal colonization in both mice and rats, as compared to the wild-type strain (ClfB^+^). In human, the mutant strain ClfB^-^ of *S. aureus* has been reported to be cleared faster than the wild-type strain (median 3 ± 1 vs. 7 ± 4 days, *p* = 0.006), whereas the wild-type strain persisted for 28 days after inoculation (Wertheim et al., 2008).

IsdA also plays a role in the adherence of *S. aureus* to nasal cells as demonstrated *in vitro* and *in vivo* (Clarke et al., 2004). Mutation in IsdA gene reduced the ability of the bacteria to bind human nasal cells *in vitro* and to colonize the anterior nares in cotton rats (Clarke et al., 2006). This surface protein has also been shown to bind to fibrinogen and fibronectin (Clarke et al., 2004). The role of IsdA in nasal colonization has not been clearly demonstrated in human studies yet.

Other *S. aureus* surface proteins, such as surface protein G (SasG), SasX, and the serine-aspartate repeat proteins SdrC and SdrD may also serve as ligands to the epithelial cells (Corrigan et al., 2009; Liu Q. et al., 2015). Desmoglein 1 was identified as a host ligand for SdrD (Askarian et al., 2016). Their roles in humans have not been tested yet (Mulcahy et al., 2012).

## Interaction in the Inner Nasal Cavity

Apart from the *vestibulum nasi*, the inner part of the nasal cavity constitutes another ecological niche for *S. aureus.* The staphylococcal nonprotein adhesin, named cell *wall teichoic acid* (WTA) is considered as an important factor for the colonization process (Weidenmaier et al., 2004). In an *in vivo* study, WTA-deficient *S. aureus* mutants could not adhere to nasal cells and were unable to colonize cotton rat nares compared to wild type control strains (Weidenmaier et al., 2004).

In a study combining *in vitro* and *in vivo* assessments, Baur et al. (2014) studied the molecular details of WTA adhesion to nasal cells. They first discovered that SREC-1 (a member of the F-type scavenger receptor) was expressed on epithelial cells in the inner nasal cavity of human and cotton rats. The authors further reported that SREC-1 interacted with WTA and confirmed these findings in infected cotton rats pretreated with antiSREC-1 antibody. A significant decrease in colonization was observed 8 h and 6 days after inoculation in the group receiving antiSREC-1 antibody, as compared to the controls (Baur et al., 2014). In agreement, Weidenmaier et al. (2008) previously demonstrated the role of WTA in the initial stages of *S. aureus* colonization.

## Intracellular Localization of *S. aureus*

Intracellular localization of *S. aureus* in the nasal tissue has been described using techniques including immunohistochemical analysis, hematoxylin, and Eosin stains (Hayes et al., 2015; Ou et al., 2016, 2017). Epithelial cells, endothelial cells, and inflammatory cells, especially mast cells (Plouin-Gaudon et al., 2006; Tan et al., 2014; Hayes et al., 2015; Ou et al., 2016) have been described as habitats for intracellular *S. aureus* (ICSA). Using skin biopsies from the *vestibulum nasi* of randomly selected healthy participants, Hanssen et al. (2017) detected intracellular *S. aureus* in the *stratum spinosum*, one of the layers forming the epidermis. Intracellular localization of *S. aureus* has been reported both in patients with rhinosinusitis (Clement et al., 2005; Plouin-Gaudon et al., 2006; Ou et al., 2016, 2017) and healthy volunteers (Hanssen et al., 2017). In the control population from the study of Ou et al. (2016), 38% had ICSA. However, in the absence of large scale population study, the true prevalence of ICSA in healthy individuals remains to determine.

Intracellular localization of *S. aureus* seems to protect the bacteria from host defense mechanisms (Clement et al., 2005) and favor antimicrobial agents’ failure, as it was demonstrated in a recent study evaluating systemic and topical antibiotics. Using a cell model of *S. aureus* nasal epithelium evasion, Rigaill et al. (2018) found that most of the decolonizing agents, including mupirocin, exhibited weak activity against ICSA. Elimination of ICSA was also studied in a mouse model using vancomycin. In this study, intracellular bacteria were able to establish infection even in the presence of this antibiotic (Lehar et al., 2015), whereas planktonic bacteria were eliminated by vancomycin and did not cause infection. The intracellular residency of *S. aureus* could explain re-colonization and failure in decolonization observed in some healthy carriers, and the recurrence of infections observed in patients with chronic rhinosinusitis (Clement et al., 2005; Ou et al., 2017; Rigaill et al., 2018).

## Immune System and *S. aureus* Colonization

Our knowledge in terms of host response during *S. aureus* nasal colonization is still limited as compared to immune response in invasive *S. aureus* infection (Brown et al., 2014). The association between the host immune system and *S. aureus* nasal carriage has been recently reviewed by Mulcahy and McLoughlin (2016). The presence of *S. aureus* in the nares seems to induce both innate and adaptive immune system. *S. aureus* can otherwise overcome host defense mechanisms. The staphylococcal protein A (SpA) and the exoprotein staphylokinase could play a key role in the bacterial adaptation to the immune system and colonization process (Peschel and Sahl, 2006; Cole et al., 2016).

### Innate Immune Response

Human nasal secretions have efficient natural antimicrobial activity (Cole et al., 1999). Indeed, they contain epithelial-cells secreted antimicrobial peptides (AMPs) contributing to the first line defense in the nose. Primary nasal epithelial cells yield AMPs such as hBD3 and RNase-7 after stimulation with cytokines (IFN-g, IL-1b, and TNF-a) (Burgey et al., 2016). Media containing *S. aureus* induce the expression of these AMPs less efficiently than inflammatory cytokines (Burgey et al., 2016). Differential expression profiles of AMPs could be primary determinants for different *S. aureus* carriage states (Burgey et al., 2016).

Anti-staphylococcal responses can be induced by the involvement of Toll-like receptors (TLR). Hence, the role of TLR2 has been documented in nasal *S. aureus* colonization (González-Zorn et al., 2005). Carrier strains of *S. aureus* have been shown to delay the host’s immune response compared to non-carrier strains due to the delayed TLR2 expression they stimulate in nasal epithelial cells (Quinn and Cole, 2007). Different polymorphisms have been found in *S. aureus* nasal carriers and non-carriers; they target soluble or membrane-binding molecules such as genes encoding mannose-binding lectin, glucocorticoid receptor, C-reactive protein, beta-defensin 1, TLR-9, and interleukin-4 (van den Akker et al., 2006; Emonts et al., 2008; Ruimy et al., 2010; Mulcahy and McLoughlin, 2016; Nurjadi et al., 2018).

A study evaluating neutrophil depletion was performed in mice to determine the importance of neutrophil influx during carriage. Mice that received an antibody for neutrophil depletion had a significant increase in *S. aureus* colonization rate as compared to controls. This *in vivo* study highlights that neutrophil influx plays a role in clearance of *S. aureus* (Archer et al., 2013).

### Adaptive Immune Response

As documented from serum analysis, *S. aureus* carriage induces an adaptive humoral immune response. Serum levels of immunoglobulin IgG and IgA specific to several staphylococcal proteins have been reported to be higher in persistent carriers than in non-carriers (van Belkum et al., 2009; Colque-Navarro et al., 2010). But evidence that these antibodies are protective against colonization are lacking.

In a murine model of nasal colonization, clearance of *S. aureus* was found to be B-cell independent but T-cell mediated. The Th17 cells, a functional T-cell lineage distinct from Th1 and Th2 are known to be involved in mucosal barriers and surface pathogen clearance. They typically produce IL-17 cytokine family. Archer et al. (2013, 2016) showed that IL-17A and IL-17F deficient mice failed to clear *S. aureus* experimentally inoculated through nasal route; IL-17A was required for AMP expression induced by nasal colonization. In a murine model, the IL-10 family cytokine IL-22 was secreted by Th17 cells and facilitated local expression of AMPs. IL-22 also reduced the expression of staphylococcal ligands loricrin and cytokeratin 10 and thus controlled nasal colonization (Mulcahy et al., 2016).

By contrast, in a human study evaluating nasal fluid inflammatory factors and nasal carriage with *S. aureus*, IL-17 was not detected (Cole et al., 2016). This was surprising and the authors suggested that IL-17 could be insoluble in nasal secretions (Cole et al., 2016). Low Th1 to Th17 cytokines ratio were found to be predictive of *S. aureus* carriage in volunteers after whole blood stimulation with *S. aureus* (Nurjadi et al., 2016). Future human studies should be conducted to better understand the role of Th17 cytokines in nasal carriage.

## Microbiology-Based Classification of Nasal Carriers

In order to confirm nasal carriage of *S. aureus*, samples are commonly collected using commercial dry or moistened sterile swabs, with no significant difference found between the two (Warnke et al., 2016). The protocol of sampling is not really standardized but generally consists of rubbing the swab in the anterior nares of each nostril for approximately four rotary movements (Wertheim et al., 2006; Bode et al., 2010). Swabs are then analyzed to check presence of *S. aureus*. The two commonly used laboratory tests for the identification of *S. aureus* are the culture on chromogenic solid media – which is the less expensive test – and polymerase chain reaction, which is the gold standard and the most rapid technique for MRSA detection (Pournajaf et al., 2014). It is important to note that chronic carriage of *S. aureus* may not only result from nasal colonization. Sampling extra-nasal sites like oropharynx, rectum, wounds, axilla increases MRSA detection rate in patients at risk for *S. aureus* nosocomial infection (Miller et al., 2012; McKinnell et al., 2013).

Epidemiological studies over periods varying from 12 weeks to 3 years have described three nasal carriage patterns for *S. aureus* among healthy volunteers swabbed several times. Persistent carriers which rates ranged from 10% to 30% (Williams, 1963; Eriksen et al., 1995; VandenBergh et al., 1999; Nouwen et al., 2004; Muthukrishnan et al., 2013), non-carriers which rates ranged from 10% to 47%, and the rest who were considered intermittent carriers (Williams, 1963; Eriksen et al., 1995; VandenBergh et al., 1999; Nouwen et al., 2004). The definitions used for this classification varied from one study to another. In some studies, persistent carriers were the ones who had results of all of their swabs positive for *S. aureus* (Muthukrishnan et al., 2013). Other studies defined cut-off values for carrier–index (number of positive swabs/number of total swabs for each person) (Eriksen et al., 1995; VandenBergh et al., 1999). However, there is no clear definition on the number of swabs that should be taken and what fraction should be positive before determining carriage state.

Nouwen et al. (2004) proposed a culture rule based on the combination of qualitative and quantitative results of two consecutive nasal culture swabs taken approximately a week apart, to predict *S. aureus* nasal carrier state among healthy volunteers. When both cultures are positive over 10^3^ colony forming units (cfu) a person is classified as persistent carrier. When only one of the cultures is positive, or when both cultures are positive with a low cfu, the person is considered as an intermittent carrier. van Belkum et al. (2009) performed a study in 51 volunteers with a known carriage state, who were artificially colonized with a mixture of *S. aureus* strains after a decolonization treatment and followed for 22 weeks. Median nasal bacterial survival was of 4 days in noncarriers, 14 days in intermittent carriers, and more than 154 days in persistent carriers (*P* = 0.017). The culture swabs of persistent carriers contained more bacterial loads compared to the other groups. Serum levels of anti-staphylococcal antibodies differed between persistent and nonpersistent carriers as previously stated. The authors concluded that intermittent and noncarriers share similar characteristics, and thus suggested a reclassification of *S. aureus* nasal carriers into persistent carriers and “other” carriers (van Belkum et al., 2009). Persistent carriers have been shown to have higher counts of *S. aureus* (Nouwen et al., 2004; van Belkum et al., 2009) and a higher risk of infection compared to other carriers (Wertheim et al., 2004; Nouwen et al., 2005). However, bacterial loads in persistent carriers are always variable and it is hard to determine a fixed threshold for diagnosis (Burian et al., 2010; Verhoeven et al., 2012; Nilsson et al., 2015). Persistent carriage is more common in children than in adults, but many people change their carrier state between 10 and 20 years old (Armstrong-Esther, 1976). Conclusions that persistent carriers harbor the same strain for many years, and intermittent carriers appear to have changing strains (Eriksen et al., 1995; VandenBergh et al., 1999) have been recently the subject of controversy (Muthukrishnan et al., 2013).

Cross-sectional studies yield a prevalence of approximately 20–30% of carriers in the general population which is a mix of persistent and intermittent carriers (Gorwitz et al., 2008; den Heijer et al., 2013; Saadatian-Elahi et al., 2013; Chen et al., 2017).

## Individual Risk Factors for *S. aureus* Nasal Colonization

Nasal colonization depends on host factors, such as the underlying condition or diseases (**Table 2**). Some studies have found that nasal carriage was more frequent in human immunodeficiency virus (HIV)-infected (Raviglione et al., 1990; Weinke et al., 1992; Kotpal et al., 2016) or obese patients (Olsen et al., 2013), compared to healthy individuals. This higher prevalence was also found among diabetic patients undergoing dialysis compared to non-diabetic patients in the same population (Luzar et al., 1990). Other diseases such as granulomatosiss with polyangiitis (formerly known as Wegener’s granulomatosis), rheumatoid arthritis (Laudien et al., 2010), skin and soft tissue infections (Immergluck et al., 2017) atopic dermatitis (Breuer et al., 2002), and recurrent furunculosis (Demos et al., 2012) have been related with an increased carriage rate.

**Table 2:** Predisposing factors for nasal carriage.

Predisposing factors for nasal carriage	Reference
HIV-infection	Raviglione et al., 1990; Weinke et al., 1992; Kotpal et al., 2016
Obesity	Olsen et al., 2013
Diabetic patients undergoing dialysis (compared to non-diabetic patients in the same population)	Luzar et al., 1990
Granulomatosis with polyangiitis	Laudien et al., 2010
Rheumatoid arthritis	Laudien et al., 2010
Skin and soft tissue infections	Immergluck et al., 2017
Recurrent furunculosis	Demos et al., 2012
Atopic dermatitis	Breuer et al., 2002
Hemoglobin in nasal secretions	Pynnonen et al., 2011
Histocompatibility antigen phenotype HLA-DR3	Kinsman et al., 1983
Polymorphisms in genes encoding for the glucocorticoid receptor, interleukin-4, C-reactive proteins, and complement inhibitor proteins	van den Akker et al., 2006; Emonts et al., 2008; Ruimy et al., 2010
Hormonal contraception use	Zanger et al., 2012
Active smokers: controversial	Olsen et al., 2012; Cole et al., 2018
Hospital workers: controversial	Elie-Turenne et al., 2010; Saadatian-Elahi et al., 2013; Chen et al., 2015; Price et al., 2017

In healthy subjects, Liu C.M. et al. (2015) found similar carriage rates in men and women, while men had higher bacterial density. Reports of a higher risk of nasal carriage of *S. aureus* among hospital workers than the rest of the population have not been confirmed (Elie-Turenne et al., 2010; Saadatian-Elahi et al., 2013; Chen et al., 2015; Price et al., 2017). The association between smoking and nasal carriage seems also controversial. In a study by Olsen et al. (2012), active smoking in healthy adults was found to be a protective factor for carriage of *S. aureus*, with a hypothesized bactericidal activity of cigarette smokes. Conversely, a recent study showed that smokers were more frequently colonized than non-smokers, and cessation from smoking improved clearance of nasal *S. aureus* in an experimental inoculation study (Cole et al., 2018). Many other host conditions have been punctually studied and reported as additional predisposing factor such as hormonal contraception (Zanger et al., 2012) and presence of hemoglobin in nasal secretions (Pynnonen et al., 2011).

At the genetic level, no correlation was found between genetic factors and *S. aureus* carriage. No significant heritability for *S. aureus* nasal colonization was detected in twins and family studies (Roghmann et al., 2011; Andersen et al., 2012). Interestingly, some polymorphisms in host inflammatory response genes have been associated with *S. aureus* nasal carriage. The presence of the histocompatibility antigen phenotype HLA-DR3 could be a predisposition (Kinsman et al., 1983).

As previously said, at the immune system level, polymorphisms in genes encoding some proteins and differential expression profiles of AMPs could be the determinants of the various carriage states.

In a study involving 93 type 1 diabetes patients, vitamin D receptor polymorphisms were determined in Deoxyribonucleic acid (DNA) extracted from peripheral blood leukocytes. Analysis showed that presence of specific alleles coding for vitamin D receptors were associated with an increased rate of *S. aureus* colonization (Panierakis et al., 2009).

## Nasal Carriage of *S. aureus* as a Risk Factor for Infections

*Staphylococcus aureus* nasal colonization has been identified as a major risk factor for the development of patent staphylococcal infections, weather community acquired, or nosocomial (Von Eiff et al., 2001; Wertheim et al., 2004, 2005b) which increases the risk by 2 to 10 times (Perl and Golub, 1998). The risk of infection in nasal carriers has been mainly studied in surgical patients (general, orthopedic, cardiac, and neurosurgeries) (Perl et al., 2002; Bode et al., 2010; Walsh et al., 2017), patients on hemodialysis (Kluytmans et al., 1996; Katneni and Hedayati, 2007), patients on chronic ambulatory peritoneal dialysis (CAPD) (Luzar et al., 1990), HIV-infected patients (Nguyen et al., 1999; Sissolak et al., 2002), and intensive care unit patients (Nardi et al., 2001). It has also been shown to be the primary risk factor for recurrent furunculosis, nasal colonization being present in almost 60% of individuals with furuncles and impetigo (Durupt et al., 2007) (**Figure 2** and **Table 3**).

**Table 3:** *S. aureus* nasal colonization, a risk factor for infections.

*S. aureus* nasal colonization, a risk factor for	Reference
Surgical site infections after orthopedic surgeries	Kalmeijer et al., 2000; Yano et al., 2000; Weiser and Moucha, 2015
Surgical site infections after cardiac surgeries	Kluytmans et al., 1995; Muñoz et al., 2008
Bacteremia in nonsurgical patients	Wertheim et al., 2004
Catheter-related infections in dialysis patients	Luzar et al., 1990; Katneni and Hedayati, 2007
*S. aureus* infections in HIV-infected patients	Nguyen et al., 1999; Sissolak et al., 2002
ICU-associated *S. aureus* infections	Honda et al., 2010
Recurrent furunculosis and impetigo	Durupt et al., 2007; Demos et al., 2012
Diabetic foot ulcer infections	Dunyach-Remy et al., 2017

## Surgical Site Infections

Developing a post-operative infection is a multifactorial process usually combining preoperative, intraoperative, and post-operative factors (Savage and Anderson, 2013). Nasal colonization can actually be considered as a preoperative risk factor for MRSA and MSSA infections. *S. aureus* can spread from the anterior nares to other areas on the skin surface and thus contaminate the surgical wound during the operative procedure (NICE Clinical Guidelines, 2008; Savage and Anderson, 2013). It has been shown that around 80% of strains causing a staphylococcal infection at the site of surgery have molecular identity with *S. aureus* isolates in the nares of concerned patients (Perl et al., 2002).

Surgical site infections (SSI) are one of the most common post-operative complications and represent 20 to 30% of healthcare associated infections (HCAI) (Klevens et al., 2007; Magill et al., 2012; Savage and Anderson, 2013). While enterobacteria and other uro-digestive bacteria are dominant in infections after gastrointestinal, urological and gynecological surgeries (Trautman et al., 2007), *S. aureus* predominates in orthopedic and cardiac surgery settings (Lepelletier et al., 2005; Trautman et al., 2007; Muñoz et al., 2008). In orthopedic patients, biofilms can form on the implants leading to therapeutic challenges (Chen et al., 2013). According to the French Institute for Public Health Surveillance in 2014, *S. aureus* composed 19.4% of isolated germs in SSIs after coronary surgeries, 51.9% of organisms causing SSIs in orthopedic surgeries, and 29.3% of organisms causing infections in gynecologic obstetric surgery in 2014 (Le Réseau d’alerte, d’investigation et de surveillance des infections nosocomiales (Raisin), 2015). Similar implications have been also found in other countries (Saadatian-Elahi et al., 2008; Negi et al., 2015).

Several studies including case–control and multivariate analysis have identified nasal carriage of *S. aureus* an independent risk factor for SSIs (Kluytmans et al., 1995; Kalmeijer et al., 2000; Muñoz et al., 2008). In the case–control study from Kluytmans et al. (1995), cardiac surgery patients were screened for their nasal carriage status the day before surgery and followed for the development of an SSI. *S. aureus* wound infections occurred in 40 patients as opposed to 120 controls who did not develop infection. Nasal carriage, identified in 52% of cases as compared to 12% of controls, was found to be a significant risk factor for the development of these post-operative infections with an OR = 9.6, 95% CI (3.9–23.7). In a study involving 357 patients undergoing major heart surgery, nasal carriers had a higher incidence of SSI than non-carriers (12.5% vs. 5%, *P* = 0.01) (Muñoz et al., 2008). Similar conclusions were obtained for orthopedic patients with an incidence increasing from 3- to 11-fold (Kalmeijer et al., 2000; Yano et al., 2000; Weiser and Moucha, 2015).

Surgical site infections have an important impact on the patient and the healthcare system. These infections increase hospital stay (Leaper et al., 2004), mortality and healthcare costs and decrease health-related quality of life (Anderson et al., 2009; Lamarsalle et al., 2013; Savage and Anderson, 2013). A retrospective database analysis in France concluded that staphylococcal infections led to approximately 1.0 and 1.4 additional hospitalizations per patient, 22.1 and 22.4 additional hospital days, and an excess cost of €15,475 and €13,389 after cardiothoracic and orthopedic surgeries respectively (Schmidt et al., 2015). The rate of in-hospital mortality was 2.6 times and six times higher among infected patients than non-infected patients in cardiothoracic and orthopedic procedures (Schmidt et al., 2015).

Methicillin-resistant *S. aureus* carriage could increase the risk for the development of SSIs. In a systematic review, patients colonized with MRSA were four times more likely to develop invasive infection than patients colonized with MSSA (Safdar and Bradley, 2008). MRSA infections are reported to cause up to 40% of HCAI worldwide with particularly high incidence in the United States and many European countries (McKinnell et al., 2013) and have been shown to have increased morbi-mortality as compared to MSSA (Anderson et al., 2009). In a study carried out by Anderson et al. (2009), patients with SSI due to MRSA had a 2.6 higher risk of dying within 3 months, the duration of hospital stay was 6 days longer, and the related cost was increased of $23,000 compared to patients with SSI due to MSSA. On the other hand, a meta-analysis of 31 cohort studies on *S. aureus* bacteremia, showed significantly higher mortality with MRSA than with MSSA (Cosgrove et al., 2003). However, the reasons for increased fatality rate with MRSA infections are unclear. Some authors suggest that MRSA and MSSA bacteria are equally virulent but MRSA infections usually develop in patients previously treated with antibiotics. Thus, as previously suggested, the differences in patients’ fatality rate may rather reflect the severity of underlying conditions than a higher bacteria-related increased virulence (Humphreys, 2012; Hraiech et al., 2013).

## Infections in Nonsurgical Patients

Nasal carriage of *S. aureus* has also been found to be a risk factor for subsequent infections in nonsurgical patients. Wertheim et al. (2004) screened 14,008 adults who had nasal swab on admission in a nonsurgical department, 3420 (24%) were positive for *S. aureus*. The follow-up identified 81 patients who developed *S. aureus* bacteremia between 2 and 120 days after swabbing, which was three times more frequent in carriers than in non-carriers. Interestingly, the death rate from *S. aureus* bacteremia was higher in non-carriers than carriers. This could be due to the protective immunity of anti-staphylococcal antibodies (Wertheim et al., 2004; Holtfreter et al., 2006). Approximately, 80% of *S. aureus* blood isolates causing bacteremia were of endogenous origins and identical to those isolated from the anterior nares of corresponding patients, thus confirming previous report (Von Eiff et al., 2001).

A prospective cohort study evaluated the occurrence of *S. aureus* infections in 5161 patients who were screened for nasal carriage when admitted to the ICU. ICU-associated *S. aureus* infections were defined by the development of infection >48 h after their admission to the unit. These infections occurred in 113 patients and nasal colonization was associated with a 2.5 to 4.7-fold increased risk (Honda et al., 2010).

In HIV-positive patients, a prospective cohort study evaluating 231 subjects every 3 months for a minimum of two years, has reported a 6% incidence of *S. aureus* infections, nasal carriers being more at risk [*p* = 0.04, OR = 3.6 (0.9–15.4)] (Nguyen et al., 1999).

In hemodialysis and chronic peritoneal dialysis patients, most of infectious complications come from endogenous origin (Luzar et al., 1990; Ena et al., 1994). *S. aureus* is the most common isolated agent from central venous catheter-related bacteremia (Katneni and Hedayati, 2007), or exit-site infections of peritoneal dialysis catheters (Luzar et al., 1990). Nasal carriers are at increased risk of contracting these infections (Nouwen et al., 2005; Ong et al., 2017).

*Staphylococcus aureus* is the most frequently isolated pathogen from diabetic foot infections. A study compared the genotypic profiles of *S. aureus* strains isolated from the nares and diabetic foot ulcer infections of 276 patients. The bacterium was isolated from both sites in 36% of the population, and identical strains were found in 65% of cases. Further investigations should be performed in order to confirm the benefit of screening and treating nasal carriers in this population (Dunyach-Remy et al., 2017).

Nasal carriage of *S. aureus* constitutes a risk factor for the development of skin and soft tissue infections caused by this germ in non-hospitalized and non-diseased subjects (Chou et al., 2015). It has been shown to be the primary risk factor for recurrent furunculosis (Demos et al., 2012). Nasal carriage is also a risk factor for secondary bacterial pneumonia in patients having influenza A virus infection. The viral infection causes host physiologic changes that generates the dissemination of *S. aureus* from nasal tissue to the lungs as demonstrated in a mouse model (Reddinger et al., 2016).

## Perspectives

Nasal carriage of *S. aureus* is multifactorial and can predispose carriers to subsequent infections. Nasal decolonization of carriers is therefore recommended in patients undergoing cardiothoracic and orthopedic surgeries (De Jonge et al., 2016).

A full understanding of host-pathogen interactions can help find new decolonization strategies. New fields on colonization mechanisms should be investigated. For example, the role of mycobiota starts to be described in the pathophysiology of chronic respiratory diseases (Brégeon and Rolain, 2015). Interaction of nasal *S. aureus* with the nasal fungal communities would be an interesting perspective to develop. On the other hand, some reports suggest that *S. aureus* can regulate host inflammatory gene expression (Modak et al., 2014). Epigenetics mechanisms could be interesting to investigate in order to better understand tolerance mechanisms in *S. aureus* colonization processes.

## Author Contributions

AS wrote the review paper. FB, J-LM, OB, and J-MR corrected the manuscript. All authors approved and revised the final version of the manuscript.

## Conflict of Interest Statement

The authors declare that the research was conducted in the absence of any commercial or financial relationships that could be construed as a potential conflict of interest.

## References

[B1] Andersen P. S. Pedersen J. K. Fode P. Skov R. L. Fowler V. G. Stegger M. (2012). Influence of host genetics and environment on nasal carriage of *Staphylococcus aureus* in danish middle-aged and elderly twins. *J. Infect. Dis.* 206 1178–1184. 10.1093/infdis/jis491 22872733 PMC3448969

[B2] Anderson D. J. Kaye K. S. Chen L. F. Schmader K. E. Choi Y. Sloane R. (2009). Clinical and financial outcomes due to methicillin resistant *Staphylococcus aureus* surgical site infection: a multi-center matched outcomes study. *PLoS One* 4:e8305. 10.1371/journal.pone.0008305 20016850 PMC2788700

[B3] Archer N. K. Adappa N. D. Palmer J. N. Cohen N. A. Harro J. M. Lee S. K. (2016). Interleukin-17A (IL-17A) and IL-17F are critical for antimicrobial peptide production and clearance of *Staphylococcus aureus* nasal colonization. *Infect. Immun.* 84 3575–3583. 10.1128/IAI.00596-16 27736775 PMC5116734

[B4] Archer N. K. Harro J. M. Shirtliff M. E. (2013). Clearance of *Staphylococcus aureus* nasal carriage is T cell dependent and mediated through interleukin-17A expression and neutrophil influx. *Infect. Immun.* 81 2070–2075. 10.1128/IAI.00084-13 23529621 PMC3676016

[B5] Armstrong-Esther C. A. (1976). Carriage patterns of *Staphylococcus aureus* in a healthy non-hospital population of adults and children. *Ann. Hum. Biol.* 3 221–227. 10.1080/03014467600001381 962302

[B6] Askarian F. Ajayi C. Hanssen A.-M. van Sorge N. M. Pettersen I. Diep D. B. (2016). The interaction between *Staphylococcus aureus* SdrD and desmoglein 1 is important for adhesion to host cells. *Sci. Rep.* 6:22134. 10.1038/srep22134 26924733 PMC4770587

[B7] Baur S. Rautenberg M. Faulstich M. Faulstich M. Grau T. Severin Y. (2014). A nasal epithelial receptor for *Staphylococcus aureus* WTA governs adhesion to epithelial cells and modulates nasal colonization. *PLoS Pathog.* 10:e1004089. 10.1371/journal.ppat.1004089 24788600 PMC4006915

[B8] Bode L. G. M. Kluytmans J. A. J. W. Wertheim H. F. L. Bogaers D. Vandenbroucke-Grauls C. M. J. E. Roosendaal R. (2010). Preventing surgical-site infections in nasal carriers of *Staphylococcus aureus*. *N. Engl. J. Med.* 362 9–17. 10.1056/NEJMoa0808939 20054045

[B9] Brégeon F. Rolain J.-M. (2015). Le résistome pulmonaire. *Méd. Sci.* 31 947–950. 10.1051/medsci/20153111003 26576596

[B10] Breuer K. HAussler S. Kapp A. Werfel T. (2002). *Staphylococcus aureus*: colonizing features and influence of an antibacterial treatment in adults with atopic dermatitis. *Br. J. Dermatol.* 147 55–61. 10.1046/j.1365-2133.2002.04872.x 12100185

[B11] Brown A. F. Leech J. M. Rogers T. R. McLoughlin R. M. (2014). *Staphylococcus aureus* colonization: modulation of host immune response and impact on human vaccine design. *Front. Immunol.* 4:507. 10.3389/fimmu.2013.00507 24409186 PMC3884195

[B12] Burgey C. Kern W. V. Römer W. Rieg S. (2016). Differential induction of innate defense antimicrobial peptides in primary nasal epithelial cells upon stimulation with inflammatory cytokines, Th17 cytokines or bacterial conditioned medium from *Staphylococcus aureus* isolates. *Microb. Pathog.* 90 69–77. 10.1016/j.micpath.2015.11.023 26616165

[B13] Burian M. Wolz C. Goerke C. (2010). Regulatory adaptation of *Staphylococcus aureus* during nasal colonization of humans. *PLoS One* 5:e10040. 10.1371/journal.pone.0010040 20386721 PMC2850373

[B14] Candi E. Schmidt R. Melino G. (2005). The cornified envelope: a model of cell death in the skin. *Nat. Rev. Mol. Cell Biol.* 6 328–340. 10.1038/nrm1619 15803139

[B15] Chang C.-H. Chen S.-Y. Lu J.-J. Chang C.-J. Chang Y. Hsieh P.-H. (2017). Nasal colonization and bacterial contamination of mobile phones carried by medical staff in the operating room. *PLoS One* 12:e0175811. 10.1371/journal.pone.0175811 28562676 PMC5450997

[B16] Chen A. F. Heyl A. E. Xu P. Z. Rao N. Klatt B. A. (2013). Preoperative decolonization effective at reducing staphylococcal colonization in total joint arthroplasty patients. *J. Arthroplasty* 28 18–20. 10.1016/j.arth.2013.03.036 23871467

[B17] Chen B. Dai X. He B. Pan K. Li H. Liu X. (2015). Differences in *Staphylococcus aureus* nasal carriage and molecular characteristics among community residents and healthcare workers at Sun Yat-sen University, Guangzhou, Southern China. *BMC Infect. Dis.* 15:303. 10.1186/s12879-015-1032-7 26223250 PMC4520063

[B18] Chen B. J. Xie X. Y. Ni L. J. Dai X. L. Lu Y. Wu X. Q. (2017). Factors associated with *Staphylococcus aureus* nasal carriage and molecular characteristics among the general population at a Medical College Campus in Guangzhou, South China. *Ann. Clin. Microbiol. Antimicrob.* 16:28. 10.1186/s12941-017-0206-0 28399856 PMC5387264

[B19] Chou Y.-H. Lee M.-S. Lin R.-Y. Wu C.-Y. (2015). Risk factors for methicillin-resistant *Staphylococcus aureus* skin and soft-tissue infections in outpatients in Taiwan. *Epidemiol. Infect.* 143 749–753. 10.1017/S0950268814001642 25703397 PMC9507104

[B20] Clarke S. R. Brummell K. J. Horsburgh M. J. McDowell P. W. Mohamad S. A. S. Stapleton M. R. (2006). Identification of *in vivo*-expressed antigens of *Staphylococcus aureus* and their use in vaccinations for protection against nasal carriage. *J. Infect. Dis.* 193 1098–1108. 10.1086/501471 16544250

[B21] Clarke S. R. Wiltshire M. D. Foster S. J. (2004). IsdA of *Staphylococcus aureus* is a broad spectrum, iron-regulated adhesin. *Mol. Microbiol.* 51 1509–1519. 10.1111/j.1365-2958.2003.03938.x 14982642

[B22] Clement S. Vaudaux P. Francois P. Schrenzel J. Huggler E. Kampf S. (2005). Evidence of an intracellular reservoir in the nasal mucosa of patients with recurrent *Staphylococcus aureus* rhinosinusitis. *J. Infect. Dis.* 192 1023–1028. 10.1086/432735 16107955

[B23] Cole A. L. Muthukrishnan G. Chong C. Beavis A. Eade C. R. Wood M. P. (2016). Host innate inflammatory factors and staphylococcal protein A influence the duration of human *Staphylococcus aureus* nasal carriage. *Mucosal Immunol.* 9 1537–1548. 10.1038/mi.2016.2 26838052 PMC4972712

[B24] Cole A. L. Schmidt-Owens M. Beavis A. C. Chong C. F. Tarwater P. M. Schaus J. (2018). Cessation from smoking improves innate host defense and clearance of experimentally inoculated nasal *S. aureus. Infect. Immun*. 86 e912–e917. 10.1128/IAI.00912-17 29311241 PMC5865040

[B25] Cole A. M. Dewan P. Ganz T. (1999). Innate antimicrobial activity of nasal secretions. *Infect. Immun.* 67 3267–3275. 10377100 10.1128/iai.67.7.3267-3275.1999 PMC116505

[B26] Colque-Navarro P. Jacobsson G. Andersson R. Flock J.-I. Möllby R. (2010). Levels of antibody against 11 *Staphylococcus aureus* antigens in a healthy population. *Clin. Vaccine Immunol.* 17 1117–1123. 10.1128/CVI.00506-09 20445005 PMC2897265

[B27] Corrigan R. M. Miajlovic H. Foster T. J. Kluytmans J. van Belkum A. Verbrugh H. (2009). Surface proteins that promote adherence of *Staphylococcus aureus* to human desquamated nasal epithelial cells. *BMC Microbiol.* 9:22. 10.1186/1471-2180-9-22 19183486 PMC2642834

[B28] Cosgrove S. E. Sakoulas G. Perencevich E. N. Schwaber M. J. Karchmer A. W. Carmeli Y. (2003). Comparison of mortality associated with methicillin-resistant and methicillin-susceptible *Staphylococcus aureus* bacteremia: a meta-analysis. *Clin. Infect. Dis.* 36 53–59. 10.1086/345476 12491202

[B29] Cunningham D. J. (1905). *Cunningham’s Text-Book of Anatomy.* New York, NY?: W. Wood and company.

[B30] Dall’Antonia M. Coen P. G. Wilks M. Whiley A. Millar M. (2005). Competition between methicillin-sensitive and -resistant *Staphylococcus aureus* in the anterior nares. *J. Hosp. Infect.* 61 62–67. 10.1016/j.jhin.2005.01.008 15893854

[B31] De Jonge S. Atema J. J. Gans S. Boermeester M. A. Gomes S. M. Solomkin J. S. (2016). Surgical site infections 1 New WHO recommendations on preoperative measures for surgical site infection prevention: an evidence-based global perspective. *Lancet Infect. Dis.* 16 e276–e287. 10.1016/S1473-3099(16)30398-X 27816413

[B32] Demos M. McLeod M. P. Nouri K. (2012). Recurrent furunculosis: a review of the literature. *Br. J. Dermatol.* 167 725–732. 10.1111/j.1365-2133.2012.11151.x 22803835

[B33] den Heijer C. D. van Bijnen E. M. Paget W. J. Pringle M. Goossens H. Bruggeman C. A. (2013). Prevalence and resistance of commensal *Staphylococcus aureus*, including meticillin-resistant *S. aureus*, in nine European countries: a cross-sectional study. *Lancet Infect. Dis.* 13 409–415. 10.1016/S1473-3099(13)70036-7 23473661

[B34] Dunyach-Remy C. Courtais-Coulon C. DeMattei C. Jourdan N. Schuldiner S. Sultan A. (2017). Link between nasal carriage of *Staphylococcus aureus* and infected diabetic foot ulcers. *Diabetes Metab.* 43 167–171. 10.1016/j.diabet.2016.09.003 27720361

[B35] Durupt F. Mayor L. Bes M. Reverdy M.-E. Vandenesch F. Thomas L. (2007). Prevalence of *Staphylococcus aureus* toxins and nasal carriage in furuncles and impetigo. *Br. J. Dermatol.* 157 1161–1167. 10.1111/j.1365-2133.2007.08197.x 17916211

[B36] Eckhart L. Lippens S. Tschachler E. Declercq W. (2013). Cell death by cornification. *Biochim. Biophys. Acta Mol. Cell Res.* 1833 3471–3480. 10.1016/j.bbamcr.2013.06.010 23792051

[B37] Elie-Turenne M.-C. Fernandes H. Mediavilla J. R. Rosenthal M. Mathema B. Singh A. (2010). Prevalence and characteristics of *Staphylococcus aureus* colonization among healthcare professionals in an urban teaching hospital. *Infect. Control Hosp. Epidemiol.* 31 574–580. 10.1086/652525 20426580

[B38] Emonts M. Uitterlinden A. G. Nouwen J. L. Kardys I. Maat M. P. M. Melles D. C. (2008). Host polymorphisms in interleukin 4 complement factor H, and C-reactive protein associated with nasal carriage of *Staphylococcus aureus* and occurrence of boils. *J. Infect. Dis.* 197 1244–1253. 10.1086/533501 18422436

[B39] Ena J. Boelaert J. R. Boyken L. D. Van Landuyt H. W. Godard C. A. Herwaldt L. A. (1994). Epidemiology of *Staphylococcus aureus* infections in patients on hemodialysis. *Infect. Control Hosp. Epidemiol.* 15 78–81. 10.2307/30145535 8201238

[B40] Eriksen N. H. Espersen F. Rosdahl V. T. Jensen K. (1995). Carriage of *Staphylococcus aureus* among 104 healthy persons during a 19-month period. *Epidemiol. Infect.* 115 51–60. 10.1017/S0950268800058118 7641838 PMC2271555

[B41] Frank D. N. Feazel L. M. Bessesen M. T. Price C. S. Janoff E. N. Pace N. R. (2010). The human nasal microbiota and *Staphylococcus aureus* carriage. *PLoS One* 5:e10598. 10.1371/journal.pone.0010598 20498722 PMC2871794

[B42] Garrouste-Orgeas M. Timsit J. F. Kallel H. Ben Ali A. Dumay M. F. Paoli B. (2001). Colonization with methicillin-resistant *Staphylococcus aureus* in ICU patients: morbidity, mortality, and glycopeptide use. *Infect. Control Hosp. Epidemiol.* 22 687–692. 10.1086/501846 11842988

[B43] Ghasemzadeh-Moghaddam H. Neela V. van Wamel W. Hamat R. A. Shamsudin M. N. Hussin N. S. C. (2015). Nasal carriers are more likely to acquire exogenous *Staphylococcus aureus* strains than non-carriers. *Clin. Microbiol. Infect.* 21 998.e1–998.e7. 10.1016/j.cmi.2015.07.006 26183299

[B44] González-Zorn B. Senna J. P. M. Fiette L. Shorte S. Testard A. Chignard M. (2005). Bacterial and host factors implicated in nasal carriage of methicillin-resistant *Staphylococcus aureus* in mice. *Infect. Immun.* 73 1847–1851. 10.1128/IAI.73.3.1847-1851.2005 15731086 PMC1064969

[B45] Gorwitz R. J. Kruszon-Moran D. McAllister S. K. McQuillan G. McDougal L. K. Fosheim G. E. (2008). Changes in the prevalence of nasal colonization with *Staphylococcus aureus* in the united states, 2001–2004. *J. Infect. Dis.* 197 1226–1234. 10.1086/533494 18422434

[B46] Haill C. Fletcher S. Archer R. Jones G. Jayarajah M. Frame J. (2013). Prolonged outbreak of meticillin-resistant *Staphylococcus aureus* in a cardiac surgery unit linked to a single colonized healthcare worker. *J. Hosp. Infect.* 83 219–225. 10.1016/j.jhin.2012.11.019 23369471

[B47] Haim M. Trost A. Maier C. J. Achatz G. Feichtner S. Hintner H. (2010). Cytokeratin 8 interacts with clumping factor B: a new possible virulence factor target. *Microbiology* 156 3710–3721. 10.1099/mic.0.034413-0 20817646

[B48] Hanssen A.-M. Kindlund B. Stenklev N. C. Furberg A.-S. Fismen S. Olsen R. S. (2017). Localization of *Staphylococcus aureus* in tissue from the nasal vestibule in healthy carriers. *BMC Microbiol.* 17:89. 10.1186/s12866-017-0997-3 28381253 PMC5382455

[B49] Harbarth S. Liassine N. Dharan S. Herrault P. Auckenthaler R. Pittet D. (2000). Risk factors for persistent carriage of methicillin-resistant *Staphylococcus aureus*. *Clin. Infect. Dis.* 31 1380–1385. 10.1086/317484 11096006

[B50] Hayes S. M. Howlin R. Johnston D. A. Webb J. S. Clarke S. C. Stoodley P. (2015). Intracellular residency of *Staphylococcus aureus* within mast cells in nasal polyps: a novel observation. *J. Allergy Clin. Immunol.* 135 1648–1651.e5. 10.1016/j.jaci.2014.12.1929 25680455

[B51] Holtfreter S. Roschack K. Eichler P. Eske K. Holtfreter B. Kohler C. (2006). *Staphylococcus aureus* carriers neutralize superantigens by antibodies specific for their colonizing strain: a potential explanation for their improved prognosis in severe sepsis. *J. Infect. Dis.* 193 1275–1278. 10.1086/503048 16586365

[B52] Honda H. Krauss M. J. Coopersmith C. M. Kollef M. H. Richmond A. M. Fraser V. J. (2010). *Staphylococcus aureus* nasal colonization and subsequent infection in intensive care unit patients: does methicillin resistance matter? *Infect. Control Hosp. Epidemiol.* 31 584–591. 10.1086/652530 20426656 PMC4154586

[B53] Hraiech S. Roch A. Lepidi H. Atieh T. Audoly G. Rolain J.-M. (2013). Impaired virulence and fitness of a colistin-resistant clinical isolate of *Acinetobacter baumannii* in a rat model of pneumonia. *Antimicrob. Agents Chemother.* 57 5120–5121. 10.1128/AAC.00700-13 23836181 PMC3811450

[B54] Human Microbiome Project Consortium (2012). Structure, function and diversity of the healthy human microbiome. *Nature* 486 207–214. 10.1038/nature11234 22699609 PMC3564958

[B55] Humphreys H. (2012). *Staphylococcus aureus*: the enduring pathogen in surgery. *Surgeon* 10 357–360. 10.1016/j.surge.2012.05.003 23079115

[B56] Immergluck L. Jain S. Ray S. Mayberry R. Satola S. Parker T. (2017). Risk of skin and soft tissue infections among children found to be *Staphylococcus aureus* MRSA USA300 carriers. *West. J. Emerg. Med.* 18 201–212. 10.5811/westjem.2016.10.30483 28210352 PMC5305125

[B57] Iwase T. Uehara Y. Shinji H. Tajima A. Seo H. Takada K. (2010). *Staphylococcus epidermidis* Esp inhibits *Staphylococcus aureus* biofilm formation and nasal colonization. *Nature* 465 346–349. 10.1038/nature09074 20485435

[B58] Kalmeijer M. D. van Nieuwland-Bollen E. Bogaers-Hofman D. de Baere G. A. (2000). Nasal carriage of *Staphylococcus aureus* is a major risk factor for surgical-site infections in orthopedic surgery. *Infect. Control Hosp. Epidemiol.* 21 319–323. 10.1086/501763 10823564

[B59] Kaspar U. Kriegeskorte A. Schubert T. Peters G. Rudack C. Pieper D. H. (2016). The culturome of the human nose habitats reveals individual bacterial fingerprint patterns. *Environ. Microbiol.* 18 2130–2142. 10.1111/1462-2920.12891 25923378

[B60] Katneni R. Hedayati S. S. (2007). Central venous catheter-related bacteremia in chronic hemodialysis patients: epidemiology and evidence-based management. *Nat. Clin. Pract. Nephrol.* 3 256–266. 10.1038/ncpneph0447 17457359

[B61] Kinsman O. S. Mckenna R. Noble W. C. (1983). Association between histocompatability antigens (HLA) and nasal carriage of *Staphylococcus aureus*. *J. Med. Microbiol.* 16 215–220. 10.1099/00222615-16-2-215 6573514

[B62] Klevens R. M. Edwards J. R. Richards C. L. Horan T. C. Gaynes R. P. Pollock D. A. (2007). Estimating health care-associated infections and deaths in U.S. hospitals, 2002. *Public Health Rep.* 122 160–166. 17357358 10.1177/003335490712200205 PMC1820440

[B63] Kluytmans J. van Belkum A. Verbrugh H. (1997). Nasal carriage of *Staphylococcus aureus*: epidemiology, underlying mechanisms, and associated risks. *Clin. Microbiol. Rev.* 10 505–520. 9227864 10.1128/cmr.10.3.505 PMC172932

[B64] Kluytmans J. A. Mouton J. W. Ijzerman E. P. Vandenbroucke-Grauls C. M. Maat A. W. Wagenvoort J. H. (1995). Nasal carriage of *Staphylococcus aureus* as a major risk factor for wound infections after cardiac surgery. *J. Infect. Dis.* 171 216–219. 10.1093/infdis/171.1.216 7798667

[B65] Kluytmans J. A. J. W. Manders M.-J. van Bommel E. Verbrugh H. (1996). Elimination of nasal carriage of *Staphylococcus aureus* in hemodialysis patients. *Infect. Control Hosp. Epidemiol.* 17 793–797. 10.2307/30141172 8985765

[B66] Kotpal R. S K. P. Bhalla P. Dewan R. Kaur R. (2016). Incidence and risk factors of nasal carriage of *Staphylococcus aureus* in HIV-infected individuals in comparison to HIV-uninfected individuals: a case–control study. *J. Int. Assoc. Provid. AIDS Care* 15 141–147. 10.1177/2325957414554005 25331220

[B67] Krismer B. Weidenmaier C. Zipperer A. Peschel A. (2017). The commensal lifestyle of *Staphylococcus aureus* and its interactions with the nasal microbiota. *Nat. Rev. Microbiol.* 15 675–687. 10.1038/nrmicro.2017.104 29021598

[B68] Lamanna O. Bongiorno D. Bertoncello L. Grandesso S. Mazzucato S. Pozzan G. B. (2017). Rapid containment of nosocomial transmission of a rare community-acquired methicillin-resistant *Staphylococcus aureus* (CA-MRSA) clone, responsible for the Staphylococcal Scalded Skin Syndrome (SSSS). *Ital. J. Pediatr.* 43:5. 10.1186/s13052-016-0323-y 28061866 PMC5217574

[B69] Lamarsalle L. Hunt B. Schauf M. Szwarcensztein K. Valentine W. J. (2013). Evaluating the clinical and economic burden of healthcare-associated infections during hospitalization for surgery in France. *Epidemiol. Infect.* 141 2473–2482. 10.1017/S0950268813000253 23445665 PMC3821401

[B70] Laudien M. Gadola S. D. Podschun R. Hedderich J. Paulsen J. Reinhold-Keller E. (2010). Nasal carriage of *Staphylococcus aureus* and endonasal activity in Wegener s granulomatosis as compared to rheumatoid arthritis and chronic Rhinosinusitis with nasal polyps. *Clin. Exp. Rheumatol.* 28 51–55. 20412703

[B71] >Le Réseau d’alerte d’investigation et de surveillance des infections nosocomiales (Raisin) (2015). *Surveillance des Infections du Site Opératoire, France 2013.* France: Institut de veille sanitaire.

[B72] Leaper D. J. van Goor H. Reilly J. Petrosillo N. Geiss H. K. Torres A. J. (2004). Surgical site infection - a European perspective of incidence and economic burden. *Int. Wound J.* 1 247–273. 10.1111/j.1742-4801.2004.00067.x 16722874 PMC7951634

[B73] Lehar S. M. Pillow T. Xu M. Staben L. Kajihara K. K. Vandlen R. (2015). Novel antibody–antibiotic conjugate eliminates intracellular *S. aureus. Nature* 527 323–328. 10.1038/nature16057 26536114

[B74] Lepelletier D. Perron S. Bizouarn P. Caillon J. Drugeon H. Michaud J.-L. (2005). Surgical-site infection after cardiac surgery: incidence, microbiology, and risk factors. *Infect. Control Hosp. Epidemiol.* 26 466–472. 10.1086/502569 15954485

[B75] Leshem E. Maayan-Metzger A. Rahav G. Dolitzki M. Kuint J. Roytman Y. (2012). Transmission of *Staphylococcus aureus* from mothers to newborns. *Pediatr. Infect. Dis. J.* 31 360–363. 10.1097/INF.0b013e318244020e 22189535

[B76] Lina G. Boutite F. Tristan A. Bes M. Etienne J. Vandenesch F. (2003). Bacterial competition for human nasal cavity colonization: role of Staphylococcal *agr* alleles. *Appl. Environ. Microbiol.* 69 18–23. 10.1128/aem.69.1.18-23.2003 12513972 PMC152380

[B77] Liu C. M. Price L. B. Hungate B. A. Abraham A. G. Larsen L. A. Christensen K. (2015). *Staphylococcus aureus* and the ecology of the nasal microbiome. *Sci. Adv.* 1:e1400216. 10.1126/sciadv.1400216 26601194 PMC4640600

[B78] Liu Q. Du X. Hong X. Li T. Zheng B. He L. (2015). Targeting surface protein SasX by active and passive vaccination to reduce *Staphylococcus aureus* colonization and infection. *Infect. Immun.* 83 2168–2174. 10.1128/IAI.02951-14 25776748 PMC4399056

[B79] Luzar M. A. Coles G. A. Faller B. Slingeneyer A. Dah G. D. Briat C. (1990). *Staphylococcus aureus* nasal carriage and infection in patients on continuous ambulatory peritoneal dialysis. *N. Engl. J. Med.* 322 505–509. 10.1056/NEJM199002223220804 2300122

[B80] Maayan-Metzger A. Strauss T. Rubin C. Jaber H. Dulitzky M. Reiss-Mandel A. (2017). Clinical evaluation of early acquisition of *Staphylococcus aureus* carriage by newborns. *Int. J. Infect. Dis.* 64 9–14. 10.1016/j.ijid.2017.08.013 28882667

[B81] Magill S. S. Hellinger W. Cohen J. Kay R. Bailey C. Boland B. (2012). Prevalence of healthcare-associated infections in acute care hospitals in Jacksonville, Florida. *Infect. Control Hosp. Epidemiol.* 33 283–291. 10.1086/664048 22314066 PMC4648350

[B82] McKinnell J. A. Huang S. S. Eells S. J. Cui E. Miller L. G. (2013). Quantifying the impact of extranasal testing of body sites for methicillin-resistant *Staphylococcus aureus* colonization at the time of hospital or intensive care unit admission. *Infect. Control Hosp. Epidemiol.* 34 161–170. 10.1086/669095 23295562 PMC3894230

[B83] Miller L. G. Eells S. J. Taylor A. R. David M. Z. Ortiz N. Zychowski D. (2012). *Staphylococcus aureus* colonization among household contacts of patients with skin infections: risk factors, strain discordance, and complex ecology. *Clin. Infect. Dis.* 54 1523–1535. 10.1093/cid/cis213 22474221 PMC3348950

[B84] Modak R. Das Mitra S. Vasudevan M. Krishnamoorthy P. Kumar M. Bhat A. V (2014). Epigenetic response in mice mastitis: role of histone H3 acetylation and microRNA(s) in the regulation of host inflammatory gene expression during *Staphylococcus aureus* infection. *Clin. Epigenetics* 6:12. 10.1186/1868-7083-6-12 25075227 PMC4114167

[B85] Mulcahy M. E. Geoghegan J. A. Monk I. R. O’Keeffe K. M. Walsh E. J. Foster T. J. (2012). Nasal colonisation by *Staphylococcus aureus* depends upon clumping factor B binding to the squamous epithelial cell envelope protein loricrin. *PLoS Pathog.* 8:e1003092. 10.1371/journal.ppat.1003092 23300445 PMC3531522

[B86] Mulcahy M. E. Leech J. M. Renauld J.-C. Mills K. H. McLoughlin R. M. (2016). Interleukin-22 regulates antimicrobial peptide expression and keratinocyte differentiation to control *Staphylococcus aureus* colonization of the nasal mucosa. *Mucosal Immunol.* 9 1429–1441. 10.1038/mi.2016.24 27007677

[B87] Mulcahy M. E. McLoughlin R. M. (2016). Host–bacterial crosstalk determines *Staphylococcus aureus* nasal colonization. *Trends Microbiol.* 24 872–886. 10.1016/j.tim.2016.06.012 27474529

[B88] Muñoz P. Hortal J. Giannella M. Barrio J. M. Rodríguez-Créixems M. Pérez M. J. (2008). Nasal carriage of *S. aureus* increases the risk of surgical site infection after major heart surgery. *J. Hosp. Infect.* 68 25–31. 10.1016/j.jhin.2007.08.010 17945393

[B89] Muthukrishnan G. Lamers R. P. Ellis A. Paramanandam V. Persaud A. B. Tafur S. (2013). Longitudinal genetic analyses of *Staphylococcus aureus* nasal carriage dynamics in a diverse population. *BMC Infect. Dis.* 13:221. 10.1186/1471-2334-13-221 23679038 PMC3673815

[B90] Nardi G. Di Silvestre A. D. De Monte A. Massarutti D. Proietti A. Grazia Troncon M. (2001). Reduction in gram-positive pneumonia and antibiotic consumption following the use of a SDD protocol including nasal and oral mupirocin. *Eur. J. Emerg. Med.* 8 203–214. 10.1097/00063110-200109000-00008 11587466

[B91] Negi V. Pal S. Juyal D. Sharma M. K. Sharma N. (2015). Bacteriological profile of surgical site infections and their antibiogram: a study from resource constrained rural setting of uttarakhand state, India. *J. Clin. Diagn. Res.* 9 DC17–DC20. 10.7860/JCDR/2015/15342.6698 26557520 PMC4625239

[B92] Nguyen M. H. Kauffman C. A. Goodman R. P. Squier C. Arbeit R. D. Singh N. (1999). Nasal carriage of and infection with *Staphylococcus aureus* in HIV-infected patients. *Ann. Intern. Med.* 130 221–225. 10.7326/0003-4819-130-3-199902020-00026 10049200

[B93] NICE Clinical Guidelines (2008). *National Collaborating Centre for Women’s and Children’s Health (UK). Preoperative phase, Surgical Site Infection: Prevention and Treatment of Surgical Site Infection.* London: National Institute for Health and Clinical Excellence.

[B94] Nilsson A. C. Janson H. Wold H. Fugelli A. Andersson K. Håkangård C. (2015). LTX-109 is a novel agent for nasal decolonization of methicillin-resistant and -sensitive *Staphylococcus aureus*. *Antimicrob. Agents Chemother.* 59 145–151. 10.1128/AAC.03513-14 25331699 PMC4291342

[B95] Nouwen J. Schouten J. Schneebergen P. Snijders S. Maaskant J. Koolen M. (2006). *Staphylococcus aureus* carriage patterns and the risk of infections associated with continuous peritoneal dialysis. *J. Clin. Microbiol.* 44 2233–2236. 10.1128/JCM.02083-05 16757626 PMC1489388

[B96] Nouwen J. L. Fieren M. W. J. A. Snijders S. Verbrugh H. A. Van Belkum A. (2005). Persistent (not intermittent) nasal carriage of*Staphylococcus aureus* is the determinant of CPD-related infections. *Kidney Int.* 67 1084–1092. 10.1111/j.1523-1755.2005.00174.x 15698449

[B97] Nouwen J. L. Optima Grafische Communicatie (2004). *Determinants, Risks & Dynamics of Staphylococcus aureus Nasal Carriage.* Doctoral thesis, Erasmus University Rotterdam Rotterdam.

[B98] Nouwen J. L. Ott A. Kluytmans-Vandenbergh M. F. Q. Boelens H. A. M. Hofman A. van Belkum A. (2004). Predicting the *Staphylococcus aureus* nasal carrier state: derivation and validation of a “culture rule”. *Clin. Infect. Dis.* 39 806–811. 10.1086/423376 15472812

[B99] Nurjadi D. Heeg K. Weber A. N. R. Zanger P. (2018). Toll-like receptor (TLR) -9 promotor polymorphisms and gene expression are associated with persistent *Staphylococcus aureus* nasal carriage. *Clin. Microbiol. Infect.* 10.1016/j.cmi.2018.02.014 [Epub ahead of print]. 29458158

[B100] Nurjadi D. Kain M. Marcinek P. Gaile M. Heeg K. Zanger P. (2016). Ratio of T-helper type 1 (Th1) to Th17 cytokines in whole blood is associated with human β-defensin 3 expression in skin and persistent *Staphylococcus aureus* nasal carriage. *J. Infect. Dis.* 214 1744–1751. 10.1093/infdis/jiw440 27651414

[B101] O’Brien L. M. Walsh E. J. Massey R. C. Peacock S. J. Foster T. J. (2002). *Staphylococcus aureus* clumping factor B (ClfB) promotes adherence to human type I cytokeratin 10: implications for nasal colonization. *Cell. Microbiol.* 4 759–770. 10.1046/j.1462-5822.2002.00231.x 12427098

[B102] Olsen K. Danielsen K. Wilsgaard T. Sangvik M. Sollid J. U. E. Thune I. (2013). Obesity and *Staphylococcus aureus* nasal colonization among women and men in a general population. *PLoS One* 8:e63716. 10.1371/journal.pone.0063716 23667661 PMC3646820

[B103] Olsen K. Falch B. M. Danielsen K. Johannessen M. Ericson Sollid J. U. Thune I. (2012). *Staphylococcus aureus* nasal carriage is associated with serum 25-hydroxyvitamin D levels, gender and smoking status. The Tromsø Staph and Skin Study. *Eur. J. Clin. Microbiol. Infect. Dis.* 31 465–473. 10.1007/s10096-011-1331-x 21811869 PMC3303067

[B104] Ong L. M. Ch’ng C. C. Wee H. C. Supramaniam P. Zainal H. Goh B. L. (2017). Risk of peritoneal dialysis-related peritonitis in a multi-racial asian population. *Perit. Dial. Int.* 37 35–43. 10.3747/pdi.2015.00141 27147287

[B105] Ou J. Bassiouni A. Drilling A. Psaltis A. J. Vreugde S. Wormald P. J. (2017). The persistence of intracellular *Staphylococcus aureus* in the sinuses: a longitudinal study. *Rhinol. J.* 55 305–311. 10.4193/Rhin16.218 28687814

[B106] Ou J. Drilling A. Singhal D. Tan N. C.-W. Wallis-Hill D. Vreugde S. (2016). Association of intracellular *Staphylococcus aureus* with prognosis in chronic rhinosinusitis. *Int. Forum Allergy Rhinol.* 6 792–799. 10.1002/alr.21758 27080195

[B107] Panierakis C. Goulielmos G. Mamoulakis D. Maraki S. Papavasiliou E. Galanakis E. (2009). *Staphylococcus aureus* nasal carriage might be associated with vitamin D receptor polymorphisms in type 1 diabetes. *Int. J. Infect. Dis.* 13 e437–e443. 10.1016/j.ijid.2009.02.012 19411183

[B108] Peacock S. J. de Silva I. Lowy F. D. (2001). What determines nasal carriage of *Staphylococcus aureus*? *Trends Microbiol*. 9 605–610. 11728874 10.1016/s0966-842x(01)02254-5

[B109] Peacock S. J. Justice A. Griffiths D. de Silva G. D. I. Kantzanou M. N. Crook D. (2003). Determinants of acquisition and carriage of *Staphylococcus aureus* in infancy. *J. Clin. Microbiol.* 41 5718–5725. 10.1128/JCM.41.12.5718-5725.2003 14662966 PMC308978

[B110] Perkins S. Walsh E. J. Deivanayagam C. C. Narayana S. V Foster T. J. Höök M. (2001). Structural organization of the fibrinogen-binding region of the clumping factor B MSCRAMM of *Staphylococcus aureus*. *J. Biol. Chem.* 276 44721–44728. 10.1074/jbc.M106741200 11568183

[B111] Perl T. M. Cullen J. J. Wenzel R. P. Zimmerman M. B. Pfaller M. A. Sheppard D. (2002). Intranasal mupirocin to prevent postoperative *Staphylococcus aureus* infections. *N. Engl. J. Med.* 346 1871–1877. 10.1056/NEJMoa003069 12063371

[B112] Perl T. M. Golub J. E. (1998). New approaches to reduce *Staphylococcus aureus* nosocomial infection rates: treating *S. aureus* nasal carriage. *Ann. Pharmacother.* 32 S7–S16. 9475834 10.1177/106002809803200104

[B113] Peschel A. Sahl H.-G. (2006). The co-evolution of host cationic antimicrobial peptides and microbial resistance. *Nat. Rev. Microbiol.* 4 529–536. 10.1038/nrmicro1441 16778838

[B114] Plouin-Gaudon I. Clement S. Huggler E. Chaponnier C. François P. Lew D. (2006). Intracellular residency is frequently associated with recurrent *Staphylococcus aureus* rhinosinusitis. *Rhinology* 44 249–254. 17216740

[B115] Pournajaf A. Ardebili A. Goudarzi L. Khodabandeh M. Narimani T. Abbaszadeh H. (2014). PCR-based identification of methicillin-resistant *Staphylococcus aureus* strains and their antibiotic resistance profiles. *Asian Pac. J. Trop. Biomed.* 4 S293–S297. 10.12980/APJTB.4.2014C423 25183100 PMC4025288

[B116] Price J. R. Cole K. Bexley A. Kostiou V. Eyre D. W. Golubchik T. (2017). Transmission of *Staphylococcus aureus* between health-care workers, the environment, and patients in an intensive care unit: a longitudinal cohort study based on whole-genome sequencing. *Lancet Infect. Dis.* 17 207–214. 10.1016/S1473-3099(16)30413-3 27863959 PMC5266793

[B117] Pynnonen M. Stephenson R. E. Schwartz K. Hernandez M. Boles B. R. (2011). Hemoglobin promotes *Staphylococcus aureus* nasal colonization. *PLoS Pathog.* 7:e1002104. 10.1371/journal.ppat.1002104 21750673 PMC3131264

[B118] Quinn G. A. Cole A. M. (2007). Suppression of innate immunity by a nasal carriage strain of *Staphylococcus aureus* increases its colonization on nasal epithelium. *Immunology* 122 80–89. 10.1111/j.1365-2567.2007.02615.x 17472720 PMC2265977

[B119] Raviglione M. C. Mariuz P. Pablos-Mendez A. Battan R. Ottuso P. Taranta A. (1990). High *Staphylococcus aureus* nasal carriage rate in patients with acquired immunodeficiency syndrome or AIDS-related complex. *Am. J. Infect. Control* 18 64–69. 10.1016/0196-6553(90)90083-5 2186669

[B120] Reagan D. R. Doebbeling B. N. Pfaller M. A. Sheetz C. T. Houston A. K. Hollis R. J. (1991). Elimination of coincident *Staphylococcus aureus* nasal and hand carriage with intranasal application of mupirocin calcium ointment. *Ann. Intern. Med.* 114 101–106. 10.7326/0003-4819-114-2-101 1898585

[B121] Reddinger R. M. Luke-Marshall N. R. Hakansson A. P. Campagnari A. A. (2016). Host physiologic changes induced by influenza a virus lead to *Staphylococcus aureus* biofilm dispersion and transition from asymptomatic colonization to invasive disease. *mBio* 7:e01235-16. 10.1128/mBio.01235-16 27507829 PMC4981728

[B122] Regev-Yochay G. Trzcinski K. Thompson C. M. Malley R. Lipsitch M. (2006). Interference between *Streptococcus pneumoniae* and *Staphylococcus aureus*: *in vitro* hydrogen peroxide-mediated killing by *Streptococcus pneumoniae*. *J. Bacteriol.* 188 4996–5001. 10.1128/JB.00317-06 16788209 PMC1482988

[B123] Rigaill J. Morgene M. F. Gavid M. Lelonge Y. He Z. Carricajo A. (2018). Intracellular activity of antimicrobial compounds used for *Staphylococcus aureus* nasal decolonization. *J. Antimicrob. Chemother.* 10.1093/jac/dky318 [Epub ahead of print]. 30124897

[B124] Roche F. M. Meehan M. Foster T. J. (2003). The *Staphylococcus aureus* surface protein SasG and its homologues promote bacterial adherence to human desquamated nasal epithelial cells. *Microbiology* 149 2759–2767. 10.1099/mic.0.26412-0 14523109

[B125] Roghmann M.-C. Johnson J. K. Stine O. C. Lydecker A. D. Ryan K. A. Mitchell B. D. (2011). Persistent *Staphylococcus aureus* colonization is not a strongly heritable trait in Amish families. *PLoS One* 6:e17368. 10.1371/journal.pone.0017368 21386985 PMC3046241

[B126] Ruimy R. Angebault C. Djossou F. Dupont C. Epelboin L. Jarraud S. (2010). Are host genetics the predominant determinant of persistent nasal *Staphylococcus aureus* carriage in humans? *J. Infect. Dis.* 202 924–934. 10.1086/655901 20677941

[B127] Saadatian-Elahi M. Teyssou R. Vanhems P. (2008). *Staphylococcus aureus*, the major pathogen in orthopaedic and cardiac surgical site infections: a literature review. *Int. J. Surg.* 6 238–245. 10.1016/j.ijsu.2007.05.001 17561463

[B128] Saadatian-Elahi M. Tristan A. Laurent F. Rasigade J.-P. Bouchiat C. Ranc A.-G. (2013). Basic rules of hygiene protect health care and lab workers from nasal colonization by *Staphylococcus aureus*: an international cross-sectional study. *PLoS One* 8:e82851. 10.1371/journal.pone.0082851 24367562 PMC3867406

[B129] Safdar N. Bradley E. A. (2008). The risk of infection after nasal colonization with *Staphylococcus aureus*. *Am. J. Med.* 121 310–315. 10.1016/j.amjmed.2007.07.034 18374690

[B130] Savage J. W. Anderson P. A. (2013). An update on modifiable factors to reduce the risk of surgical site infections. *Spine J.* 13 1017–1029. 10.1016/j.spinee.2013.03.051 23711958

[B131] Schaffer A. C. Solinga R. M. Cocchiaro J. Portoles M. Kiser K. B. Risley A. (2006). Immunization with *Staphylococcus aureus* clumping factor B, a major determinant in nasal carriage, reduces nasal colonization in a murine model. *Infect. Immun.* 74 2145–2153. 10.1128/IAI.74.4.2145-2153.2006 16552044 PMC1418917

[B132] Schmidt A. Bénard S. Cyr S. (2015). Hospital cost of staphylococcal infection after cardiothoracic or orthopedic operations in France: a retrospective database analysis. *Surg. Infect.* 16 428–435. 10.1089/sur.2014.045 26207403 PMC4523037

[B133] Selva L. Viana D. Regev-Yochay G. Trzcinski K. Corpa J. M. Lasa I. (2009). Killing niche competitors by remote-control bacteriophage induction. *Proc. Natl. Acad. Sci. U.S.A.* 106 1234–1238. 10.1073/pnas.0809600106 19141630 PMC2633583

[B134] Sherertz R. J. Reagan D. R. Hampton K. D. Robertson K. L. Streed S. A. Hoen H. M. (1996). A cloud adult: the *Staphylococcus aureus*-virus interaction revisited. *Ann. Intern. Med.* 124 539–547. 10.7326/0003-4819-124-6-199603150-00001 8597316

[B135] Sissolak D. Geusau A. Heinze G. Witte W. Rotter M. L. (2002). Risk factors for nasal carriage of *Staphylococcus aureus* in infectious disease patients, including patients infected with HIV, and molecular typing of colonizing strains. *Eur. J. Clin. Microbiol. Infect. Dis.* 21 88–96. 10.1007/s10096-001-0666-0 11939405

[B136] Sivaraman K. Venkataraman N. Cole A. M. (2009). *Staphylococcus aureus* nasal carriage and its contributing factors. *Future Microbiol.* 4 999–1008. 10.2217/fmb.09.79 19824791 PMC2908500

[B137] Steinert P. M. Marekov L. N. (1995). The proteins elafin, filaggrin, keratin intermediate filaments, loricrin, and small proline-rich proteins 1 and 2 are isodipeptide cross-linked components of the human epidermal cornified cell envelope. *J. Biol. Chem.* 270 17702–17711. 10.1074/jbc.270.30.17702 7543090

[B138] Sugimoto S. Iwamoto T. Takada K. Okuda K.-I. Tajima A. Iwase T. (2013). *Staphylococcus epidermidis* Esp degrades specific proteins associated with *Staphylococcus aureus* biofilm formation and host-pathogen interaction. *J. Bacteriol.* 195 1645–1655. 10.1128/JB.01672-12 23316041 PMC3624567

[B139] Tan N. C.-W. Cooksley C. M. Roscioli E. Drilling A. J. Douglas R. Wormald P.-J. (2014). Small-colony variants and phenotype switching of intracellular *Staphylococcus aureus* in chronic rhinosinusitis. *Allergy* 69 1364–1371. 10.1111/all.12457 24922342

[B140] Trautman M. Stecher J. Luz K. Hemmer W. Hupp T. Grützner P. A. (2007). The use of mupirocin in the eradication of *Staphylococcus aureus*. *Dtsch. Arztebl.* 104:A-3259.

[B141] Uehara Y. Nakama H. Agematsu K. Uchida M. Kawakami Y. Abdul Fattah A. S. M. (2000). Bacterial interference among nasal inhabitants: eradication of *Staphylococcus aureus* from nasal cavities by artificial implantation of *Corynebacterium* sp. *J. Hosp. Infect.* 44 127–133. 10.1053/jhin.1999.0680 10662563

[B142] van Belkum A. Verkaik N. J. de Vogel C. P. Boelens H. A. Verveer J. Nouwen J. L. (2009). Reclassification of *Staphylococcus aureus* nasal carriage types. *J. Infect. Dis.* 199 1820–1826. 10.1086/599119 19419332

[B143] van den Akker E. L. T. Nouwen J. L. Melles D. C. van Rossum E. F. C. Koper J. W. Uitterlinden A. G. (2006). *Staphylococcus aureus* nasal carriage is associated with glucocorticoid receptor gene polymorphisms. *J. Infect. Dis.* 194 814–818. 10.1086/506367 16941349

[B144] VandenBergh M. F. Yzerman E. P. van Belkum A. Boelens H. A. Sijmons M. Verbrugh H. A. (1999). Follow-up of *Staphylococcus aureus* nasal carriage after 8 years: redefining the persistent carrier state. *J. Clin. Microbiol.* 37 3133–3140. 10488166 10.1128/jcm.37.10.3133-3140.1999 PMC85511

[B145] Verhoeven P. O. Grattard F. Carricajo A. Lucht F. Cazorla C. Garraud O. (2012). An algorithm based on one or two nasal samples is accurate to identify persistent nasal carriers of *Staphylococcus aureus*. *Clin. Microbiol. Infect.* 18 551–557. 10.1111/j.1469-0691.2011.03611.x 21851484

[B146] Von Eiff C. Becker K. Machka K. Stammer H. Peters G. (2001). Nasal carriage as a source of *Staphylococcus aureus* bacteremia. Study group. *N. Engl. J. Med.* 344 11–16. 10.1056/NEJM200101043440102 11136954

[B147] Vonberg R.-P. Stamm-Balderjahn S. Hansen S. Zuschneid I. Rüden H. Behnke M. (2006). How often do asymptomatic healthcare workers cause methicillin-resistant *Staphylococcus aureus* outbreaks? A systematic evaluation. *Infect. Control Hosp. Epidemiol.* 27 1123–1127. 10.1086/507922 17006821

[B148] Vuyk H. D. Watts S. J. (2006). “Nasal reconstruction,” in *Facial Plastic and Reconstructive Surgery* Vuyk H. D. Lohuis P. J. F. M. (London: Hodder Arnold) 455–480.

[B149] Walsh T. L. Querry A. M. McCool S. Galdys A. L. Shutt K. A. Saul M. I. (2017). Risk factors for surgical site infections following neurosurgical spinal fusion operations: a case control study. *Infect. Control Hosp. Epidemiol.* 38 340–347. 10.1017/ice.2016.307 27989249

[B150] Wang J.-T. Chang S.-C. Ko W.-J. Chang Y.-Y. Chen M.-L. Pan H.-J. (2001). A hospital-acquired outbreak of methicillin-resistant *Staphylococcus aureus* infection initiated by a surgeon carrier. *J. Hosp. Infect.* 47 104–109. 10.1053/jhin.2000.0878 11170773

[B151] Warnke P. Devide A. Weise M. Frickmann H. Schwarz N. G. Schäffler H. (2016). Utilizing moist or dry swabs for the sampling of nasal MRSA carriers? An *in vivo* and *in vitro* study. *PLoS One* 11:e0163073. 10.1371/journal.pone.0163073 27626801 PMC5023121

[B152] Weidenmaier C. Goerke C. Wolz C. (2012). *Staphylococcus aureus* determinants for nasal colonization. *Trends Microbiol.* 20 243–250. 10.1016/j.tim.2012.03.004 22494802

[B153] Weidenmaier C. Kokai-Kun J. F. Kristian S. A. Chanturiya T. Kalbacher H. Gross M. (2004). Role of teichoic acids in *Staphylococcus aureus* nasal colonization, a major risk factor in nosocomial infections. *Nat. Med.* 10 243–245. 10.1038/nm991 14758355

[B154] Weidenmaier C. Kokai-Kun J. F. Kulauzovic E. Kohler T. Thumm G. Stoll H. (2008). Differential roles of sortase-anchored surface proteins and wall teichoic acid in *Staphylococcus aureus* nasal colonization. *Int. J. Med. Microbiol.* 298 505–513. 10.1016/j.ijmm.2007.11.006 18221914

[B155] Weinke T. Schiller R. Fehrenbach F. J. Pohle H. D. (1992). Association between *Staphylococcus aureus* nasopharyngeal colonization and septicemia in patients infected with the human immunodeficiency virus. *Eur. J. Clin. Microbiol. Infect. Dis.* 11 985–989. 10.1007/BF01967787 1295767

[B156] Weiser M. C. Moucha C. S. (2015). The current state of screening and decolonization for the prevention of *Staphylococcus aureus* surgical site infection after total hip and knee arthroplasty. *J. Bone Joint Surg. Am.* 97 1449–1458. 10.2106/JBJS.N.01114 26333741 PMC7535098

[B157] Wertheim H. F. L. Melles D. C. Vos M. C. van Leeuwen W. van Belkum A. Verbrugh H. A. (2005a). The role of nasal carriage in *Staphylococcus aureus* infections. *Lancet Infect. Dis.* 5 751–762. 10.1016/S1473-3099(05)70295-4 16310147

[B158] Wertheim H. F. L. Verveer J. Boelens H. A. M. van Belkum A. Verbrugh H. A. Vos M. C. (2005b). Effect of mupirocin treatment on nasal, pharyngeal, and perineal carriage of *Staphylococcus aureus* in healthy adults. *Antimicrob. Agents Chemother.* 49 1465–1467. 10.1128/AAC.49.4.1465-1467.2005 15793127 PMC1068605

[B159] Wertheim H. F. L. van Kleef M. Vos M. C. Ott A. Verbrugh H. A. Fokkens W. (2006). Nose picking and nasal carriage of *Staphylococcus aureus*. *Infect. Control Hosp. Epidemiol.* 27 863–867. 10.1086/506401 16874648

[B160] Wertheim H. F. L. Vos M. C. Ott A. van Belkum A. Voss A. Kluytmans J. A. J. W. (2004). Risk and outcome of nosocomial *Staphylococcus aureus* bacteraemia in nasal carriers versus non-carriers. *Lancet* 364 703–705. 10.1016/S0140-6736(04)16897-9 15325835

[B161] Wertheim H. F. L. Walsh E. Choudhurry R. Melles D. C. Boelens H. A. M. Miajlovic H. (2008). Key role for clumping factor B in *Staphylococcus aureus* nasal colonization of humans. *PLoS Med.* 5:e17. 10.1371/journal.pmed.0050017 18198942 PMC2194749

[B162] Williams R. E. (1963). Healthy carriage of *Staphylococcus aureus*: its prevalence and importance. *Bacteriol. Rev.* 27 56–71. 14000926 10.1128/br.27.1.56-71.1963 PMC441169

[B163] Wollenberg M. S. Claesen J. Escapa I. F. Aldridge K. L. Fischbach M. A. Lemon K. P. (2014). *Propionibacterium*-produced coproporphyrin III induces *Staphylococcus aureus* aggregation and biofilm formation. *mBio* 5:e01286-14. 10.1128/mBio.01286-14 25053784 PMC4120196

[B164] Yan M. Pamp S. J. Fukuyama J. Hwang P. H. Cho D.-Y. Holmes S. (2013). Nasal microenvironments and interspecific interactions influence nasal microbiota complexity and *S. aureus* carriage. *Cell Host Microbe* 14 631–640. 10.1016/j.chom.2013.11.005 24331461 PMC3902146

[B165] Yano M. Doki Y. Inoue M. Tsujinaka T. Shiozaki H. Monden M. (2000). Preoperative intranasal mupirocin ointment significantly reduces postoperative infection with *Staphylococcus aureus* in patients undergoing upper gastrointestinal surgery. *Surg. Today* 30 16–21. 10.1007/PL00010040 10648077

[B166] Zanger P. Nurjadi D. Gaile M. Gabrysch S. Kremsner P. G. (2012). Hormonal contraceptive use and persistent *Staphylococcus aureus* nasal carriage. *Clin. Infect. Dis.* 55 1625–1632. 10.1093/cid/cis778 22955426

[B167] Zipperer A. Konnerth M. C. Laux C. Berscheid A. Janek D. Weidenmaier C. (2016). Human commensals producing a novel antibiotic impair pathogen colonization. *Nature* 535 511–516. 10.1038/nature18634 27466123

